# Private practice dentists’ conceptions of overtreatment: A qualitative study from Norway

**DOI:** 10.2340/aos.v83.42269

**Published:** 2024-11-05

**Authors:** Julie Skrede Edvinsen, Bjørn Hofmann

**Affiliations:** a Dental Hygienist, University of Oslo; b Center for Medical Ethics, Faculty of Medicine, University of Oslo, Oslo, Norway; c Department of Health Sciences, Faculty of Medicine and Health Sciences, Norwegian University of Science and Technology (NTNU), Gjøvik, Norway

**Keywords:** Ethics, working life, self-employed, contractor, employee, working environment

## Abstract

**Objective:**

As overtreatment has gained attention and is a threat to sustainable healthcare, the objective of this study is to investigate Norwegian private practice dentists’ conceptions of overtreatment.

**Material and Methods:**

Six private practice dentists were interviewed. Interviews were transcribed verbatim and analyzed by thematic analysis in a six-step process including coding and identifying main- and sub-themes.

**Results:**

The main themes identified were conceptions of overtreatment, internal factors, and external conditions of importance for overtreatment. Norwegian private practice dentists are familiar with the concept overtreatment and provide several examples of overtreatment. Although they see overtreatment as a problem, they express that the boundaries of what is considered necessary or professionally justified treatment have changed over time – particularly towards aesthetic and cosmetic treatment. Overtreatment is considered to be less problematic if the patients are informed and consent. The participants point to several internal factors and external conditions furthering overtreatment: professional status and prestige, general social trends, social media, demographic changes, overcapacity, and the expansion of commercial chains. The dentists in the interviews demonstrated that they are aware of their power, but also acknowledge their responsibility.

**Conclusion:**

Private practice dentists in Norway are aware of overtreatment and their drivers. They acknowledge their power to promote overtreatment, but also that this gives them responsibility. This raises important issues about dentists’ professional accountability and integrity.

**Main message:**

- Dentists are aware of overtreatment
- They have many examples and consider it to be a problem and identify several internal factors and external conditions that foster overtreatment
- At the same time, they believe that overtreatment can be justified when patients want the treatment and consent, even if the treatment is not medically indicated
- This raises questions about dentists’ accountability and integrity.

## Introduction

Overactivity has been in focus in healthcare recently, and it is estimated that between 20% and 50% of healthcare services are waste [1–5]. This concern has been shared in dentistry [6–16]. In Norway, the number of practicing dentists has increased at the same time as the dental health of the population has improved [17]. There is increased competition and market thinking, which can be drivers of overactivity [18].

Results from a recently published survey show that almost 40% of all private dentists stated that they had too few patients and that they compensated for this by increasing the call-in frequency and increasing rates [19]. This demonstrates that dentists have market power and that this can result in overactivity.

To prevent overactivity in dental care, we need more in-depth knowledge of dentists’ conceptions and knowledge of, and attitudes to overtreatment. This study therefore seeks answer to the following question: *What understanding of, experiences with, and attitudes towards the phenomenon of overtreatment do privately practicing dentists have?*

## Material and method

To answer the question, a semi-structured in-depth interview has been used [20]. An interview guide was developed which was pilot tested on a dentist and revised in line with the feedback.

Participants were recruited from private clinics in Oslo, who were invited through inquiries with information letters. Six participants approached the first author and joined the study. The first author performed the interviews.

Interviews were performed at the University of Oslo and in the clinics. Audio recordings were made which were transcribed verbatim and then analyzed by using thematic analysis. This was done by abstracting meaning from data by identifying themes within the interview following a six-step process described in [21]: (1) Becoming familiar with the data, (2) generating initial codes, (3) searching for themes, (4) going through the themes, (5) defining themes and (6) summarizing the results. Unlike other qualitative research methods thematic analysis does not have one specific theoretical foundation and a reflexive thematic analysis is performed according to [21].

Both authors read and analyzed the transcribed interviews. The first author generated the initial codes (2), searched for (3) and defined (4) themes in discussion with the corresponding author. See Appendix for the Consolidated criteria for reporting qualitative studies (COREQ. 32-item checklist).

### Ethics

All participants were informed orally and in writing and signed a consent form.

Audio recordings and transcriptions were stored on a secure server for the University of Oslo, UiO (TSD). Transcribed interviews were stored anonymously.

## Results

Six dentists participated in the study. All worked in a private dental health care, had variable pay based on performance and were self-employed. Three were men and three were women and they had between two and 15 years of work experience. Some also had experience from public dental health care. All were educated in Norway.

**Figure 1:**
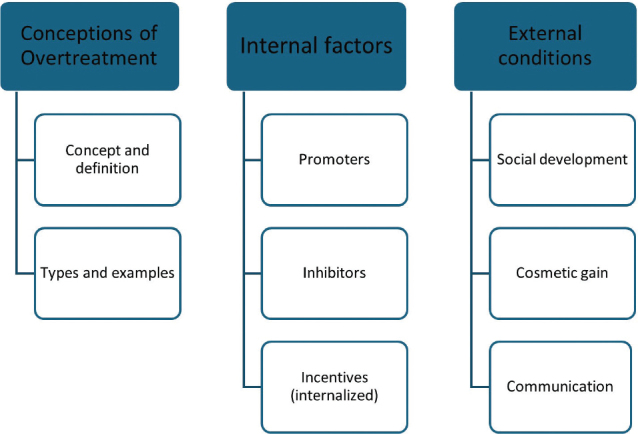
Overview of identified main themes and sub-themes.

After coding and searching by theme, as well as structuring these, the following main themes were identified: *conceptions of overtreatment, internal factors and external conditions of importance for overtreatment.* The main themes and sub-themes are shown in Figure 1.

### Conceptions of overtreatment

The participants varied in their conception of overtreatment and gave a variety of examples.

#### Concepts and definitions

The participants conceived overtreatment as medical treatment without an indication, and treatment that was not desired by the patient. One of the participants defined overtreatment as follows: ‘treatment that is not medically indicated or something that in a way is not really necessary, especially if it is not from the patient’s perspective…’ (I1). Some also distinguished between unconscious and deliberate overtreatment referring to the first as ‘overtreatment due to a lack of knowledge … [and] overtreatment due to gains other than what is in the patient’s best interest …’ (I2) and the second as ‘conscious overtreatment where you choose to take treatment that you could not, or which is not in the patient’s best interest, or not a sufficient indication for. Either financial gain or whatever else it may be’ (I2, but also I3 and I4).

Several of the participants also considered treatment without illness to be overtreatment (such as ‘changing a filling just because it is old’ and aesthetic treatment) but emphasized that such treatment is based on the patient’s own wishes and needs. Some also underlined the psychological effect and social function of aesthetic treatment, which meant that overtreatment (without health professional benefit) could be justified. Some talked about cosmetic benefits: ‘You must help patients, both with function, chewing function, but also social function…’ (I2, but also I6).

#### Types and examples

The participants also gave a wide range of examples of potential overtreatment, such as caries treatment that can be prevented prophylactically, wisdom teeth extraction, replacement of fillings, bite splints, aligners, edge-bonding and implants (I1,3,4).

### Internal factors

Participants pointed to several internal factors of overtreatment, such as fear of losing patients and financial profit, misinterpretation of X-rays, and diagnostic uncertainty. One of the participants put it this way: overtreatment can be caused by seeing a ‘shadow on an X-ray that can be interpreted incorrectly and starting treatment… that you think about turnover, increased salary… are afraid of losing the patient… the patient will move, and you know there’s going to be a cavity in 1 year … better I take the turnover’ (I2).

#### Promoters

Participants also reported pressure from managers and social media towards increased overtreatment. One participant expressed it this way: ‘I have felt under pressure to shortening the recall interval … they [managers] have shown me X-rays where they think it is caries 3, but I think it is 2 … the pressure to do treatment.3’ And that I ‘have to change a filling that is worn out’ (I3). Furthermore, the participants expressed that professional status and prestige influenced the treatment pattern: they conveyed that they ‘… would like to do a lot of crowns and bridges because it is exciting… also invisible orthodontics, getting people to look good, it has also become a bit of status… If you love doing perio is somehow not as high status as doing large implant bridges, and there is more money in it …’ (I6).

#### Inhibitors

At the same time, the participants also showed an awareness of avoiding overactivity. As one of the participants stated: ‘it is our job as dentists to put our foot down when someone who does not have a dental professional background is to interfere in the professional and beyond operations… We are the ones who decide what needs to be done’ (I5). They also emphasized the importance of professional integrity and attitudes among colleagues (in the team) and the importance of professional attention, such as the Choosing Wisely campaign, which is professional initiative to address overuse (https://www.choosingwisely.org/). Some were also afraid of getting a bad professional reputation if they became too liberal. Participants also expressed that they were aware of overtreatment in their daily practice and that they felt responsible for the treatment they provide, and that health-considerations should not be mixed up with financial issues. As one of the participants (I1) expressed it: ‘…it’s our job as dentists to put our foot down when someone who doesn’t have a dental professional background gets involved in the professional and beyond operations…It’s us who decide what needs to be done’. Participants also mentioned the Norwegian version of the Choosing Wisely Campaign as an inspiration and the importance of education in order to foster professional integrity. The importance of professional integrity was for example expressed in the need to ‘be critical when choosing a place to work, feel whether you think things are good or not. Don’t be convinced by others to do things … but stand up to individuals who … don’t dare to say against…those who just want to make money’ (I5).

#### Incentives

The respondents also reported internalized incentives, such as few patients, pay-per-performance systems, for example by suggesting various types of treatments. They also expressed fear of a bad reputation if they did not provide what patients requested, but also that it could harm dentists’ reputation if overtreatment was revealed, for example, in the media. As one participant (I2) said: ‘As a practitioner, it is very important that we have the patient’s trust… the trust between the dental profession and the patient group is something we must pay attention to… when cases of overtreatment are in newspapers or the media, it is something we dislike … setting us in a bad light’. They felt that broad or aggressive marketing (by others) influenced their own attitude and motivation.

In addition, the participants pointed to the incentives for overactivity, such as an ‘empty agenda’, commission pay, competition for patients, chain formation with economists in management and aggressive marketing. They described the development of large chains as an important factor for over-processing. As one of the participants expressed it: ‘many chains and a lot of money being thrown into the private dental health service. There are large companies that buy large chains, now we are talking about large international companies … who invest because it is a safe income for them, and then the value of a chain increases, because of the many patients a chain has and how stable its incomes are over time’ (I4).

### External conditions

Several external conditions were pointed out to have importance for overtreatment.

#### Social development

Participants pointed out that external conditions, such as the general trends in society, influenced what was perceived as important and correct treatment. In particular, the participants pointed out that social media has a great influence on patients and dentists and has contributed to blurring the line between medical and cosmetic treatment. Dentists are affected by more patients requesting the same thing. As one of the participants (I5) expressed it: ‘social media and the things you are influenced by mean that the boundary between what is medical and what is more just cosmetic treatment is whispered out a lot with the fact that many people have beautiful, fine teeth that you see on social media … as a dentist you are influenced by that, you want the patient to be satisfied with what they come up with, there are also more people who want that … then you like to do more of it’.

#### Cosmetic gain

In addition, it was also pointed out that appearance was easy to sell and that the dentist itself has great influence: ‘it is the type of treatment that is the easiest to sell … if you point out that you could benefit from a bit of whitening, it is also something they have never thought about, but then suddenly they go and think about it and become very conscious of it … we are superficial people, we want to look good …’ (I1).

The participants expressed that they noticed that the general dental health had improved in the population and that the focus therefore changed to other and less urgent problems. They acknowledged that there was increasing overtreatment today. They were particularly concerned about patients who had received extensive overtreatment abroad of poor quality. The participants also pointed out that overtreatment was not new, but that much of the activity from the past was seen as overtreatment today, such as preventive amalgam fillings and removal of wisdom teeth.

#### Communication

Several participants also pointed out that lack of communication with colleagues could stimulate overactivity. They did not know the standards of treatment of colleagues, which made it difficult to draw the line, especially if the patients demanded specific unnecessary treatment. They also emphasized the need to be cautious about how to communicate about colleagues’ work (I5).

Moreover, several participants were conscious about how they communicated with patients, for example, how they presented findings (I1, I2) and avoiding phrases such as ‘now you need to …’ (I6).

## Discussion

This study shows that the participants were familiar with the concept of overtreatment and had several examples of it occurring. Although most saw overtreatment as a problem, they also expressed that the boundaries of what was considered necessary or professionally justified treatment had changed – particularly towards aesthetic and cosmetic treatment. They believed that overtreatment was less problematic if the patients were informed and consented. The participants pointed to several internal factors and external conditions that function as drivers of overtreatment: professional status and prestige, general societal development, social media, demographic changes, overcapacity, and chain formation. While the interviews demonstrated that the private dentists acknowledged the power they had to promote overtreatment, they also expressed that they had a responsibility. The participants were familiar with the ‘guidance for good clinical practice’ (www.helsedirektoratet.no) but thought that it was not always followed stringently.

The findings in this study largely agree with other studies [6–14]. Overtreatment is recognized in Norway, as in many other countries. The conceptions of overtreatment also align with findings in other studies. The same goes for the internal factors and external conditions influencing overtreatment. However, the dental health services are organized differently and the density of dentists differs somewhat from other countries. Therefore, it is important to provide knowledge about overtreatment in different contexts.

An important finding is that dentists perceive that several services are not medically justified, but that patients’ wishes, and consent nonetheless legitimize these services. At the same time, dentists are aware that they have great opportunities to influence what patients want and agree to. This places a special responsibility on dentists.

This is a qualitative study, so the results are not intended to be generalizable. However, they provide important insight in a specific context and deeper understanding of conditions and factors that are recognizable in other settings and important for dealing with overtreatment [22, 23].

Clearly, the number of participants is limited. However, key informants convey information from many professionals, as their answers are not primarily based on their idiosyncratic opinions, but on the professional conceptions they have and gain in their professional community. Hence, the study can provide important insight into private dentists’ conceptions of overactivity [24].

Moreover, while saturation is a contested concept in thematic analysis [25], the identified themes were mentioned by several of the participants several times. Hence, there is information redundancy in the interview data. By including more participants, we may have uncovered other experiences and nuances, as practices are different. However, there are good reasons to believe that our participants were able to convey the main aspects.

All persons interviewed practiced in a city where the density of therapists is higher than in rural areas. This may mean that they are more familiar with overtreatment than their colleagues in more remote places. However, this study was not about the extent of overtreatment, but the private dentists’ conceptions and experiences. Accordingly, it is appropriate to interview persons who are most strongly exposed to the phenomenon. Moreover, as dental chains are establishing in smaller places, the findings in this study may also be relevant for less urban areas.

As researchers we reflected on our preconceptions and discussed our conceptual biases. One of us has been working in dental care, but not worked with the concept of overtreatment, while the other has worked extensively with the concept and practice of medical overactivity, but not in dental care.

The themes identified are neither exhaustive nor exclusive. As the results indicate, there is overlap between them. External conditions (such as societal developments and social media) affect the professional environment internally. Nevertheless, it may be important to distinguish between how overtreatment is perceived, internal factors and external conditions, among other things to delineate responsibility.

The participants express that improved dental health can be an important driver of overtreatment and, overall, an increased number of dentists could also contribute to this. How this will develop in the future, when an aging population will be able to increase the need for dental health services [26], is uncertain and could affect overtreatment.

## Conclusion

This study demonstrates that private practice dentists in Norway are familiar with the concept of overtreatment and have several examples of it occurring. Although they see overtreatment as a problem, they express that the boundaries of what is considered necessary or professionally justified treatment have shifted – particularly towards aesthetic and cosmetic treatment.

The study points to several internal factors and external conditions that stimulate overtreatment: professional status and prestige, general societal development, social media, demographic changes, overcapacity, and chain formation. These drivers are important in the effort to reduce unwanted overtreatment.

The study also shows that overtreatment is perceived as less problematic if the patients are informed and consent. Dentists have considerable power to promote overtreatment, but it also gives them responsibility. The study accentuates the general issue of how dentists can and should take care of this responsibility in a situation where there are greater demands for earnings, but fewer patients per dentist.

## Supplementary Material

Private practice dentists’ conceptions of overtreatment: A qualitative study from Norway

## Declarations

The manuscript is sent exclusively to you, has not been previously published elsewhere, and is not currently under review elsewhere.
