# Supplementary material for: Private practice dentists’ conceptions of overtreatment: A qualitative study from Norway
Source: Acta Odontol Scand. 2024 Nov 5;83:42269. doi: 10.2340/aos.v83.42269 (PMC11633033; doi:10.2340/aos.v83.42269)
Supplement: Private practice dentists’ conceptions of overtreatment: A qualitative study from Norway [file AOS-83-42269-s1.pdf]

Supplementary material has been published as submitted. It has not been copyedited or typeset by Acta Odontologica Scandinavica.

Consolidated criteria for reporting qualitative studies (COREQ): 32-item checklist

No. Item	Guide questions/description	Reported on Page #
**Domain 1: Research team and reflexivity**		
Personal Characteristics		
1. Inter viewer/facilitator	Which author/s conducted the interview or focus group?	First author, reported on p.3
2. Credentials	What were the researcher's credentials? E.g. PhD, MD	Dental hygienist, MSc, PhD, reported on p.1
3. Occupation	What was their occupation at the time of the study?	Dental hygienist, Ma-student, professor, p.1
4. Gender	Was the researcher male or female?	Female and Male, p.1
5. Experience and training	What experience or training did the researcher have?	Student under supervision and experienced researcher, p.1
Relationship with participants		
6. Relationship established	Was a relationship established prior to study commencement?	No, p.3
7. Participant knowledge of the interviewer	What did the participants know about the researcher? e.g. personal goals, reasons for doing the research	No, p.3
8. Interviewer characteristics	What characteristics were reported about the inter viewer/facilitator? e.g. Bias, assumptions, reasons and interests in the research topic	The interviewer had observed discussions on the topic at her workplace and wanted to explore the topic. P.3

**Domain 2: study design**		
Theoretical framework		
9. Methodological orientation and Theory	What methodological orientation was stated to underpin the study? e.g. grounded theory, discourse analysis, ethnography, phenomenology, content analysis	Thematic analysis, identifying themes within the interview data. Unlike other qualitative research methods thematic analysis does not have one theoretical foundation. In this study a reflexive thematic analysis is performed. P.3.
Participant selection		
10. Sampling	How were participants selected? e.g. purposive, convenience, consecutive, snowball	P.3
11. Method of approach	How were participants approached? e.g. face-to-face, telephone, mail, email	Information letters, p.3
12. Sample size	How many participants were in the study?	6. P.3
13. Non-participation	How many people refused to participate or dropped out? Reasons?	P.3
Setting		
14. Setting of data collection	Where was the data collected? e.g. home, clinic, workplace	Interviews were performed at the University of Oslo and in the clinics. P.3
15. Presence of non-participants	Was anyone else present besides the participants and researchers?	No.
16. Description of sample	What are the important characteristics of the sample? e.g. demographic data, date	P.3
Data collection		
17. Interview guide	Were questions, prompts, guides provided by the authors? Was it pilot tested?	Yes. P.3
18. Repeat interviews	Were repeat inter views carried out? If yes, how many?	No.
19. Audio/visual recording	Did the research use audio or visual recording to collect the data?	Yes. P.3
20. Field notes	Were field notes made during and/or after the inter view or focus group?	No.
21. Duration	What was the duration of the inter views or focus group?	30-60 min
22. Data saturation	Was data saturation discussed?	Yes. P.6
23. Transcripts returned	Were transcripts returned to participants for comment and/or correction?	No.

**Domain 3: analysis and findings**		
Data analysis		
24. Number of data coders	How many data coders coded the data?	One with supervision.
25. Description of the coding tree	Did authors provide a description of the coding tree?	Yes. See page 4.
26. Derivation of themes	Were themes identified in advance or derived from the data?	From data.
27. Software	What software, if applicable, was used to manage the data?	NA
28. Participant checking	Did participants provide feedback on the findings?	No
Reporting		
29. Quotations presented	Were participant quotations presented to illustrate the themes/findings? Was each quotation identified? e.g. participant number	Yes
30. Data and findings consistent	Was there consistency between the data presented and the findings?	Yes
31. Clarity of major themes	Were major themes clearly presented in the findings?	Yes
32. Clarity of minor themes	Is there a description of diverse cases or discussion of minor themes?	Yes
